# Chromatic Information and Feature Detection in Fast Visual Analysis

**DOI:** 10.1371/journal.pone.0159898

**Published:** 2016-08-01

**Authors:** Maria M. Del Viva, Giovanni Punzi, Steven K. Shevell

**Affiliations:** 1 NEUROFARBA Dipartimento di Neuroscienze, Psicologia, Area del Farmaco e Salute del Bambino Sezione di Psicologia, Università di Firenze, Via di San Salvi, 12 Complesso di San Salvi, Padiglione 26 – 50135, Firenze, Italy; 2 Institute for Mind & Biology, University of Chicago, 940 East 57th Street, Chicago, IL, 60637, United States of America; 3 Dipartimento di Fisica “E. Fermi” Università di Pisa, via Buonarroti, 2 56127, Pisa, Italy; 4 Istituto Nazionale di Fisica Nucleare, 56127, Pisa, Italy; 5 Fermi National Laboratory, Batavia, IL, 60510, United States of America; 6 Department of Psychology, University of Chicago, Chicago, IL, United States of America; University College London, UNITED KINGDOM

## Abstract

The visual system is able to recognize a scene based on a sketch made of very simple features. This ability is likely crucial for survival, when fast image recognition is necessary, and it is believed that a primal sketch is extracted very early in the visual processing. Such highly simplified representations can be sufficient for accurate object discrimination, but an open question is the role played by color in this process. Rich color information is available in natural scenes, yet artist's sketches are usually monochromatic; and, black-and-white movies provide compelling representations of real world scenes. Also, the contrast sensitivity of color is low at fine spatial scales. We approach the question from the perspective of optimal information processing by a system endowed with limited computational resources. We show that when such limitations are taken into account, the intrinsic statistical properties of natural scenes imply that the most effective strategy is to ignore fine-scale color features and devote most of the bandwidth to gray-scale information. We find confirmation of these information-based predictions from psychophysics measurements of fast-viewing discrimination of natural scenes. We conclude that the lack of colored features in our visual representation, and our overall low sensitivity to high-frequency color components, are a consequence of an adaptation process, optimizing the size and power consumption of our brain for the visual world we live in.

## Introduction

The flow of information provided by the visual system is immense, and an accurate interpretation of the visual scene on the shortest possible timescale, for survival purposes [1], requires large capacity and energy consumption [2–5]. However, it is well known that the visual system is capable of accurate recognition of visual scenes based on very simplified drawings. It has been shown that the visual system imposes a particularly severe data reduction at an early stage [6–7], to form a compact primal-sketch-like representation [8], allowing quick detection of the most important features in the surrounding world. Such sketches most often have been represented and discussed with only monochromatic information [8–9]. Natural scenes, however, have extensive chromatic content that is a rich source of potentially useful information, particularly high spatial-frequency chromatic information [10]. This study investigates the potential usefulness of color features, and the role they actually play in the fast recognition of objects in visual scenes.

Given the demand for fast analysis of large amounts of data, the implicit constraints of a biological system with finite capacity cannot be ignored. We have therefore tried to address the problem based on a model of early vision inspired by the architecture of artificial devices used for high-speed real-time data reduction in experimental high-energy physics (HEP)—a completely different field that happens to be subject to strong computational constraints similar to vision [9].

Several past studies have explored the mechanisms of fast vision at different scales and stimulus durations, finding that both coarse and fine spatial information are simultaneously used in fast categorization of images [11–12]. Some of these models build a bottom-up saliency map, based on concurrent simultaneous processing of color with other modalities at multiple spatial scales, that is used to drive visual attention to potentially interesting image locations [13–16]. In the first step of these models, visual input is decomposed into sets of different topographic feature maps (color, motion, orientation etc) at various scales. Within each map, spatial locations compete for saliency, and subsequently these conspicuity single-modality maps are summed into a single master saliency-map.

Each of these parallel processes require a certain amount of computing power; however, the required amount varies greatly amongst scales and modalities, and implementation details might not be necessarily the same for each of them. Computational limitations are expected to play the most important role in determining the features analyzed at the finest visible spatial scales, even more so for color. This is a direct consequence of the properties of the Fourier transform, where the information content is proportional to the square of spatial frequency. We therefore use our model, that is focused on computational cost, as a tool to explore the question of the potential role of fine-scale, information-rich color features in a context of competition among various types of available information for use by a limited capacity resource. While other studies have explored the role of color in fast vision either in complete images [17–18] or at coarse spatial scale [19], it is not clear that there is any role of color at the finer scales, and this is the focus of the present work. We perform a direct comparison of human performance based on different types of information, under the same basic assumptions on the type of data-reduction occurring in early vision processing, that are at the base of our previous work [9]:

1. The system works by keeping only the information in an image that matches some predefined local patterns, dropping everything else.
2. There is a limit to the total number of patterns that the system is able to recognize.
3. There is a limit to the average number of patterns per image that can be processed (this reflects the presence of an information bottleneck at the output of the system).
4. The system is optimized to preserve the maximum amount of information (entropy).

These assumptions lead to a unique procedure of image data reduction by extracting simplified “sketches”, that are input to all further processing. Sketched obtained from pure-luminance images proved to have the same discriminating power of original unfiltered natural images in psychophysical tests [9]. We take the same assumptions as a starting point for the investigations in this paper. Our aim is to evaluate the relative merits of luminance and color information as carriers of information in sketches of natural images, prepared under equal computational constraints. We then directly measure on human subjects the corresponding discrimination performance.

## Results and Discussion

**Fig 1:**
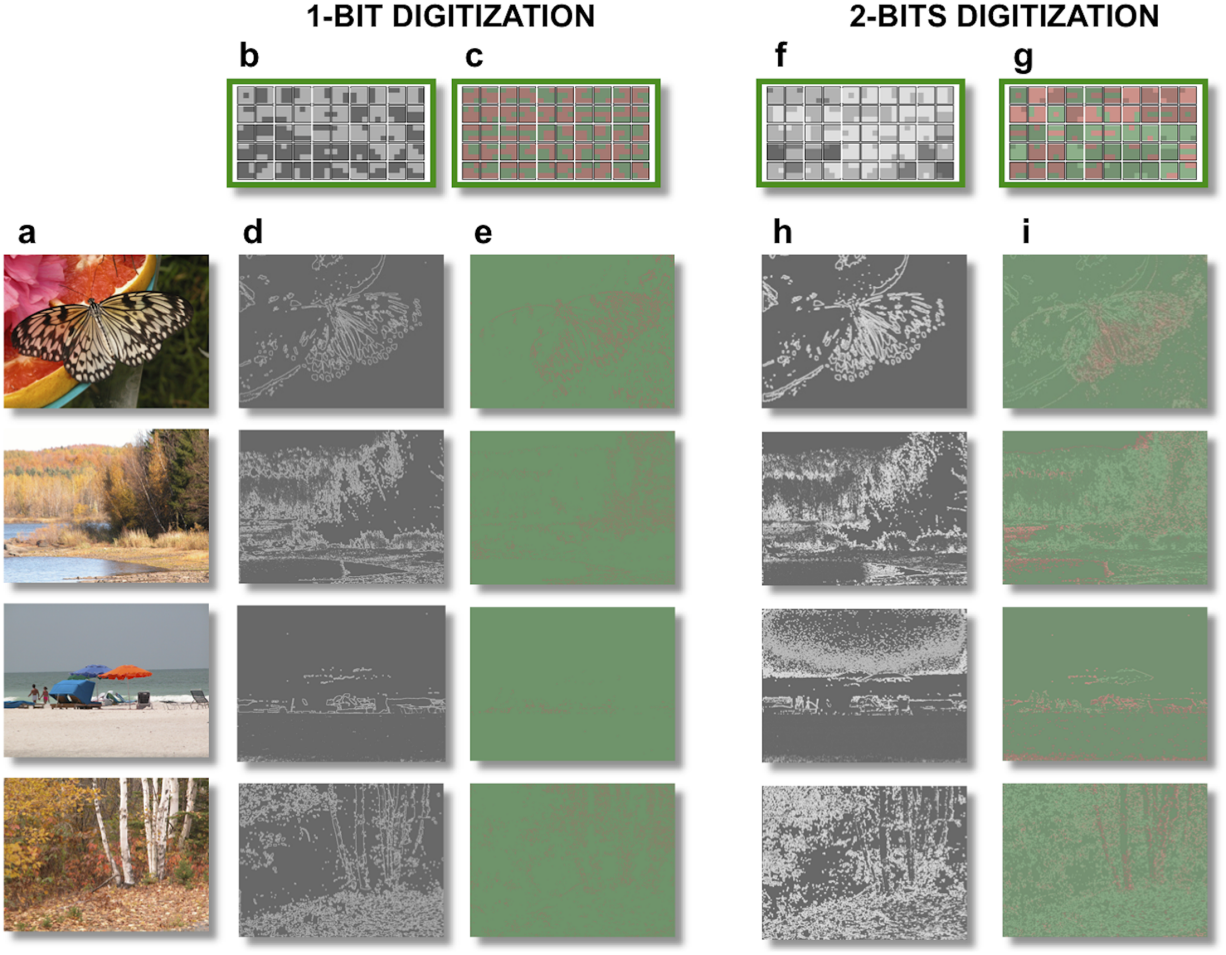
Stimuli and visual filters. a) Examples of RGB natural color images from the database. b) Set of N = 50 filters obtained after digitizing to 1 bit the *luminance* (L+M) coordinate. The actual bandwidth occupancy of the set ($\frac{1}{N_{tot}}\int_{f(p)>c}^{}p\delta (p)dp$) turns out to be slightly higher (0.06) than the imposed limit W (0.05). c) Set of N = 50 filters obtained after digitizing to one bit the *l* (L/(L+M)) coordinate. The actual bandwidth occupancy of the set out to be slightly higher (0.06) than the imposed limit W (0.05), but is still the same as the actual bandwidth of the 1 bit luminance set. d) Two gray-levels luminance sketches obtained with the filters in (b). e) Color-only sketches obtained with the filters in (c). f) Set of N = 50 filters obtained after digitizing to 2 bit the *luminance* coordinate. The actual bandwidth occupancy of the set turns out to be slightly higher (0.07) than the imposed limit W (0.05). g) Set of N = 50 filters obtained after digitizing to 1 bit both *l* (L/(L+M)) and *luminance* (L+M) coordinates. The actual bandwidth occupancy of the set turns out to be slightly higher (0.07) than the imposed limit W (0.05), but is still the same as the actual bandwidth of the 2 bits luminance set. h) Four gray-levels luminance sketches obtained the set of filters in (f). i) Color sketches obtained with the set of filters in (**g**).

Natural images were selected from a public online database [20] (Fig 1). In order to study the separate contributions of color and luminance information, the chromaticities in each image were represented in a McLeod-Boynton cone-based color space [21]. This space separates chromatic (L-cone /M-cone and S-cone) from achromatic (luminance) responses, as it happens in the retina [22].

Prior than any computation, we operate an initial reduction of information by digitizing the full resolution images (24 bits) to just 1 or 2 bits. The need for such strong reduction of levels is a corollary of the central idea of compression by pattern filtering: the number of possible patterns, that we assume to be a limited resource, increases exponentially with the number of allowed levels (that is 2n*N where n is the number of bits and N the number of pixels)–and so the amount of computing needed to calculate them. This issue is even more severe for color information, which carries three times more bits than an achromatic luminance image. Therefore, using large number of levels in our model is not only unpractical, but defeats its very purpose of saving computational resources. It is important to add that this does not amount to an important limitation for applications within the field of fast vision: the frequency of neuronal discharge is limited to ~500 Hz, which means that in 20 msec. only a very small number of spikes (~3–4) can physically be transmitted over each individual axon. Considering that we are using pretty small pixels (close to the resolution limit), this means that a very limited number of spikes are available to encode the intensity level of each signal. Even under ideal conditions, this very fact already limits the available information to very few bits.

A set of N 3x3 pixel “patterns” (feature filters) at a fine spatial scale (2 minutes of arc/pixel) was then chosen to deliver the largest possible entropy flow within the imposed limitations of bandwidth (W) and the number (N) of distinct patterns (see Methods). Patterns of this size have been previously demonstrated to be still visually discernible by normal human subjects [9]. A compressed version of each image (“sketch”) was then constructed, by filtering the image with these patterns and dropping all remaining information (Fig 1).

In the first experiment, sketches containing just one bit of color information (*L/(L+M)* in McLeod Boynton space) were compared to 1-bit luminance sketches (*L+M*).

**Fig 2:**
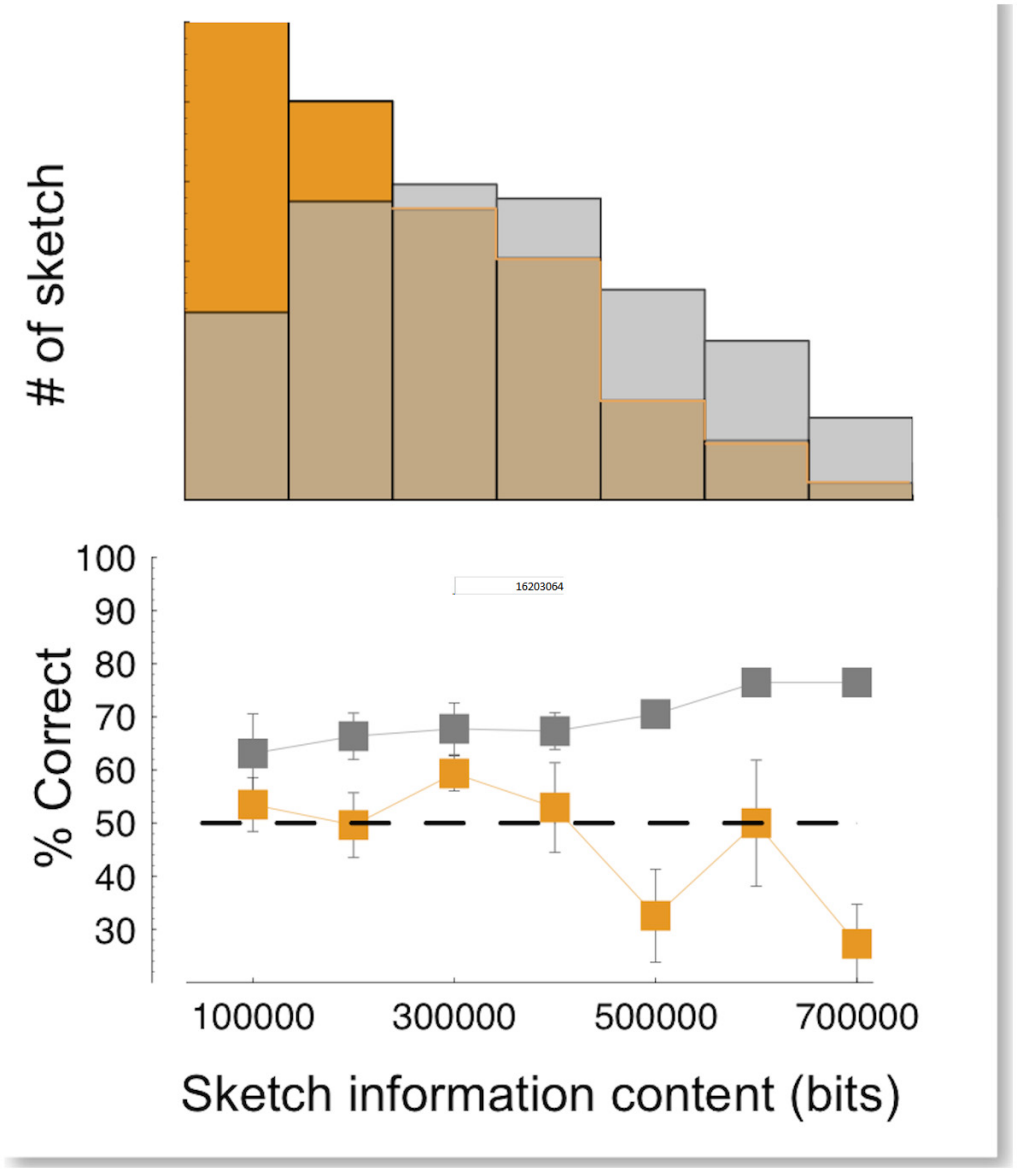
Information content of 1 bit sketches. Upper panel: distributions of the information content of the 1 bit color (orange) and luminance (gray) sketches. Information of a single sketch, S(*i*, *j*), is calculated as $\sum_{i,j}Log_{2}p(k)$, *where k* (*i*,*j*) is the pattern matching the patch centered at pixel (*i*,*j*), summed over all the image. The distributions are taken over the entire image database of our study. Lower panel: percentage of correct discrimination, averaged over all subjects, plotted as a function of the information content of the color only (orange) and luminance (gray) sketches, for the same data as in Fig 3. Error bars are SEM. The dashed line represents chance level.

Computations revealed that the average information preserved in our image set by the pure-luminance filters is greater than by pure-color filters, their distributions being shown in the upper panel of Fig 2. Since the same output capacity constraint was imposed in both conditions, this difference is entirely due to the statistical properties of the natural images represented by the human trichromatic system. Thus color features turn out to be less effective in conveying information than luminance features when a strong compression is imposed.

**Fig 3:**
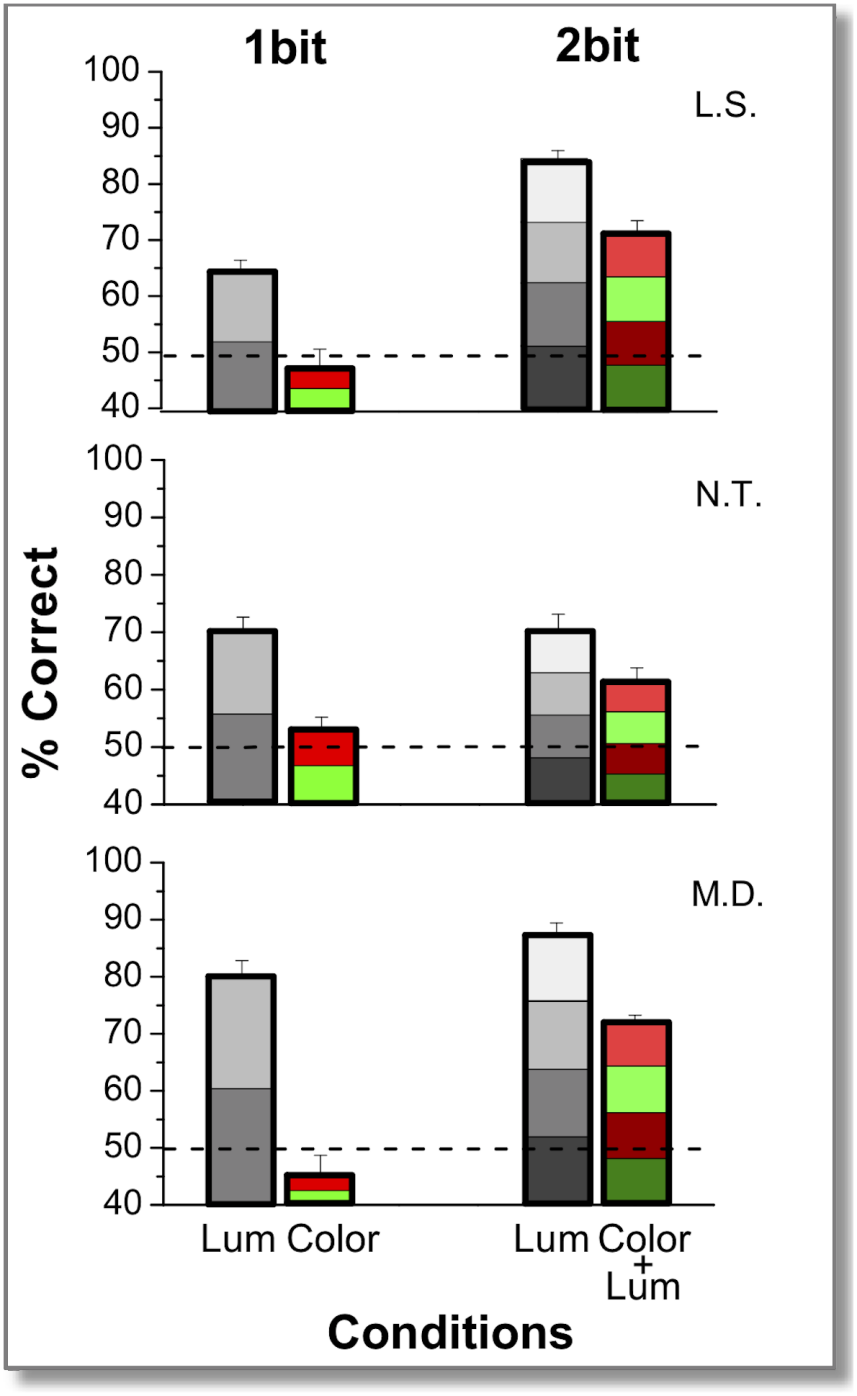
Discrimination of images based on luminance and color sketches. Percentage of correct discrimination of three subjects in four different conditions tested: 1bit luminance-only sketches (2 gray-levels bars), 1 bit equiluminant sketches (2 red/green-levels bars), 2 bit luminance-only sketches (4 gray-levels bars) and 1 bit equi-luminance + 1 bit luminance sketches (4 red/green-levels bars). Values correspond to averages over different sessions (300 trials) of the same condition. Errors are binomial s.d. The dashed line represents chance performance. *Statistical binomial tests*. 1bit *luminance* vs. 1bit *equiluminance*: *p<0*.*0001* for each subject; *equiluminance* vs. *chance p>0*.*1*; 1 bit *luminance* vs. 1 bit *equiluminance +*1 bit *luminance*: N.T and L.S *p>0*.*05*, M.D *p = 0*.*048*;1 bit *luminance* vs. 2 bits *luminance*: and L.S *p<0*.*0001*, N.T and M.D *p>0*.*1*.

To test how this difference in information content reflects onto the visual discriminability of the images, a psychophysical study of image discrimination was performed based on these sketches, presented under fast viewing conditions to probe the early stages of visual analysis [23–24]. Subjects were asked to identify which of two images corresponded to a previously briefly presented sketch, using a 2AFC procedure. The results showed that 1-bit gray-scale sketches yielded very good discriminability of the original images that was far above chance (Fig 3), in agreement with the previous study [9], despite the fact that the luminance contrast was much lower (here 30%, vs. 100% in the earlier work). Color-only (equiluminant) sketches, on the other hand, yielded far worse discrimination than luminance-only sketches, consistent with chance performance, hence failing to show any evidence that the subjects were able to use color-only information for a fast discrimination task. It is interesting to underline that these results cannot be understood simply on the basis of the lower average information content of our color sketches. The graph of Fig 2 (upper panel) shows that the distribution of information content of the two types of sketches, although different on average, is largely overlapping. This allows to plot the average discriminating performance of the subjects, separated in classes of images having the same information content, for the two type of sketches (Fig 2 lower panel). Inspection of this plot shows clearly that the response of the human visual system to the two types of sketches is completely different, even when they are compared on the basis of equivalent information content. For color sketches, the discriminability is compatible with chance level, independently of the information content. For luminance-based sketches, the discriminability is constantly above-chance over the whole range. Interestingly, the latter show a hint of increasing behavior (Pearson’s correlation coefficient r = 0.953, p = 0.00045), that is absent in the case of color sketches (Pearson’s correlation coefficient r = −0.682, p = 0.046).

All of the above observations are compatible with the visual system having made a well-defined choice in favor of using luminance-based features, and ignoring color-based features. These behavioral results are consistent with a human visual system that, under the pressure for optimization to use limited resources, follows the maximum-entropy principle. Maximum-entropy, together with natural image statistics, dictates that luminance information is the privileged vehicle for quick image recognition, at the expense of other potential sources of information.

This, however, does not exclude the possibility of color information being an important complementary source in addition to luminance information. To test for this possibility, a further experiment compared images constructed from 1 luminance bit and an additional color bit (2 bits in all) to images with 2 bits (4 levels) of luminance-only information. In spite of the fact that the compression requirements of the bottleneck were the same in both of these conditions, the entropy in the output sketches was, again, very different for the two conditions: luminance-only sketches contained, on average, 2.3 times more information than color plus luminance sketches. Corresponding psychophysical results (Fig 3) showed that the addition of 1 color bit to the luminance bit did not lead to a reliable increase of performance over the use of 1 luminance bit alone. This is consistent with the findings of Oliva and Schyns [19] where the addition of color to luminance does not significantly improve performance for spatial frequencies above 4 cycles/degree.

**Fig 4:**
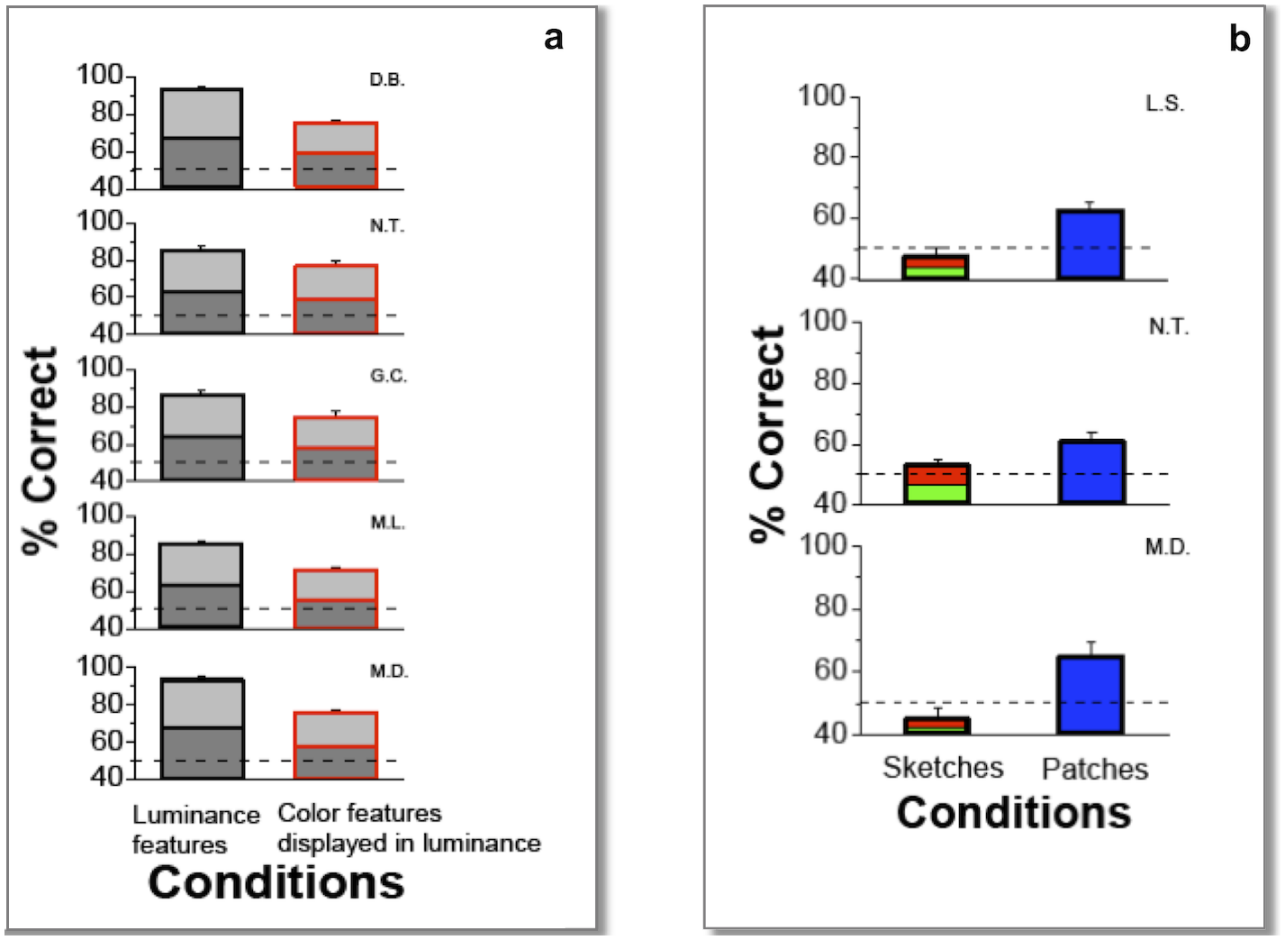
**a**) **Luminance features vs. color features rendered in luminance.** Percentage of correct discrimination of five subjects based on 2 gray-level sketches obtained with 1 bit luminance-only filters (Fig 1b) (black-outlined bars) and 1 bit color-only filters (Fig 1c) rendered in gray-levels (red outlined bars). Plotted values are averages over all trials (300) of the same condition. Errors are binomial s.d. The dashed line represents chance performance. Statistical binomial tests: N.T. and G.C: *p< 0*.*05*, D.B., M.L. and M.D. *p<0*.*01*. **b**) **Color features vs. color patches.** Percentage of correct image discrimination based on equiluminant sketches (2 red/green-levels bars) and percentage correct discrimination of color patches (blue bars). Data collected on three subjects. Plotted values are averages over all trials (300) of the same condition. Errors are binomial s.d. The dashed line represents chance performance. Color patches vs. chance: binomial tests *p<0*.*005* all subjects.

The results so far suggest a strong preference for luminance-based features over color-based features under fast visual analysis. A possible explanation for the psychophysical results might however be that the baseline physiological mechanisms underlying color perception give weaker responses than those processing luminance stimuli. That is, the chromatic information *in the image* might be useful for discrimination, but presenting that information *to the eye* chromatically might cause it to be delivered inefficiently to this fast processing system [25]. To address this question, a further experiment was conducted in which *all* image sketches were rendered in gray-scale (luminance-only differences), regardless of whether the rendered image was originally representing luminance, or color variations. In this way, the discriminability of sketches obtained from 1-bit color filters (Fig 1c) has been measured again with grayscale-rendered versions of those same sketches. These results can be compared on equal grounds with those previously obtained from the 1-bit luminance filters. Any observed difference can only be ascribed to intrinsic differences in the distribution of achromatic or chromatic features in natural images. Results still showed significantly better discrimination from sketches obtained from luminance filters than from sketches from color filters, although both were rendered in luminance (Fig 4a). We conclude that the observed differences in discrimination are dependent on the intrinsic aspects of the statistical distribution of features in natural images—that is, they reflect an intrinsic difference in the distributions of features in luminance and color, to which, presumably, the human visual system has been optimized to respond. This conclusion goes along well with previous studies indicating that high spatial-frequency chromatic information, although abundant in natural scenes [10], does not appear to be transmitted through the low-pass spatial-frequency selective pathways of the human visual system [25]. This mismatch between the spatial frequency content of the natural world and human chromatic contrast sensitivity may have the same natural explanation provided by the model, that successfully predicts the detailed structure of luminance features [9]. That is, the principle of entropy maximization under fixed computational limitations leads to suppression of fine-scale color information, because of its lower efficiency in carrying information with respect to luminance. In the framework of early vision models where multiple concurrent maps of different scales are envisioned to be processed in parallel [13–15], this conclusion means that the resolution of the color maps does not go up to the maximum resolution of the luminance maps, in order to save computational cost.

In the framework of bottom-up saliency models based on features tuned cells, where a local competition between different types of features is assumed [26–27], our results suggest that the use of color features is altogether avoided, leaving just the small number of base optimal luminance features to be recognized and accounted for in the competition process.

**Fig 5:**
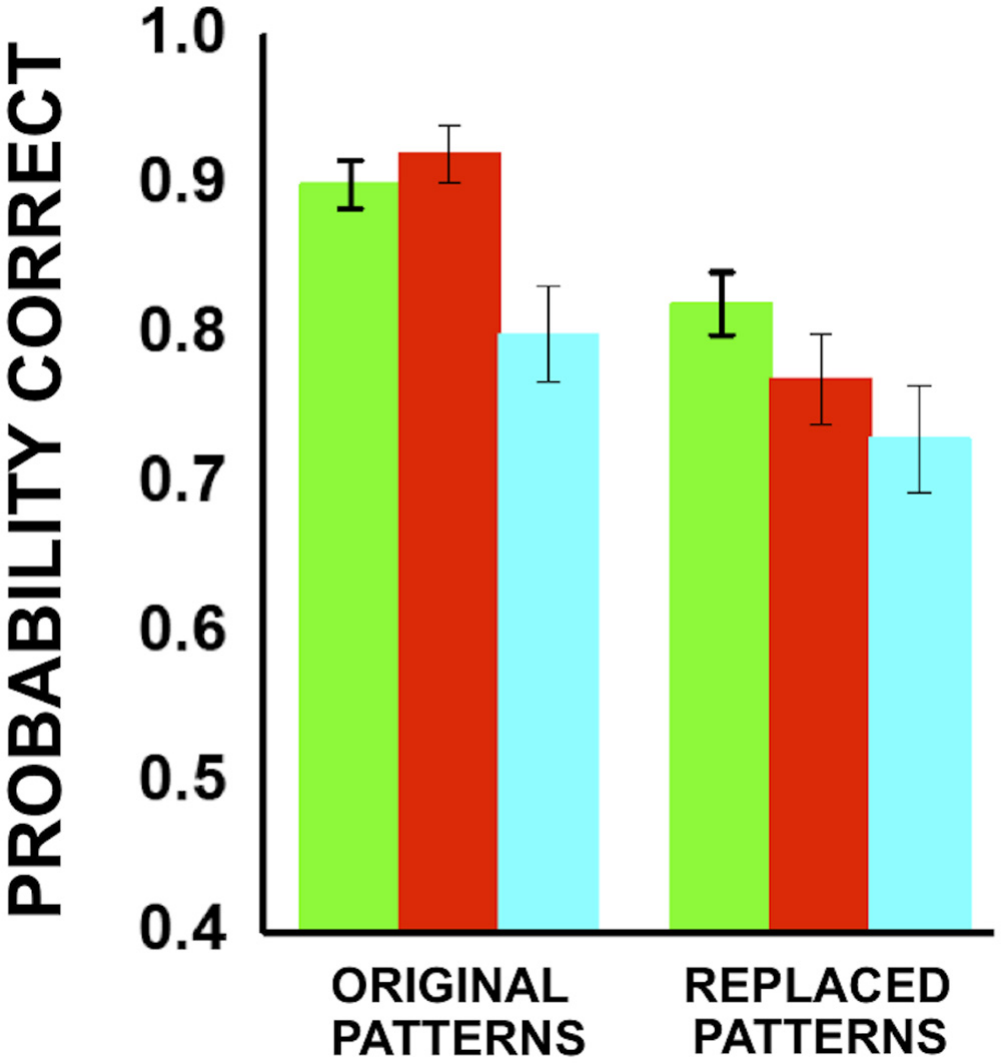
Effect of local features. Discrimination of sketches obtained with optimal patterns (left) and of sketches where optimal patterns were replaced by randomly chosen non-optimal patterns (right). Each color represents a different subject.

One might question whether under these fast-viewing conditions it is possible to see colors at all, given that it has been shown, for example, that color is processed more slowly than orientation [28]. It might be that color information is not even registered at the lowest receptor level, at the given temporal frequencies and experimental conditions. Some studies have indeed shown that color can improve discrimination of briefly presented natural stimuli, although the evidence is mixed [17, 18, 29]. To check color visibility in our specific experimental conditions, fast discriminability of uniformly-colored large stimuli was measured as a control, and was found to be well above chance, and much higher than for equiluminant sketches (Fig 4b). The existence of this color discrimination at low spatial scales confirms that color information is made available somewhere in the early levels of the visual system, and a different, finer neural architecture could in principle have been built to discriminate finer scales. Thus, the failure to discriminate equiluminant sketches is not due to a general inability to see color in fast viewing conditions, but instead to a specific lack of sensitivity to chromatic features at fine spatial scales, which should therefore be considered as nothing else than a “design choice” in the structuring of the visual system. It is worth contrasting this with what happens with luminance-only information, where the fine structure of the image seems indeed to contribute significantly to the global percept. This effect can be seen by replacing the patterns in our sketches with other randomly-chosen, non-salient patterns, keeping their localization in the image unchanged. Although the replacement cannot be practically realized at the 100% level due the unwanted appearance of new salient patterns spuriously created by the replacement process itself, it is observed that this disruption of local features causes a decrease of recognizability of the image, in spite of its global structure being preserved (Fig 5).

**Fig 6:**
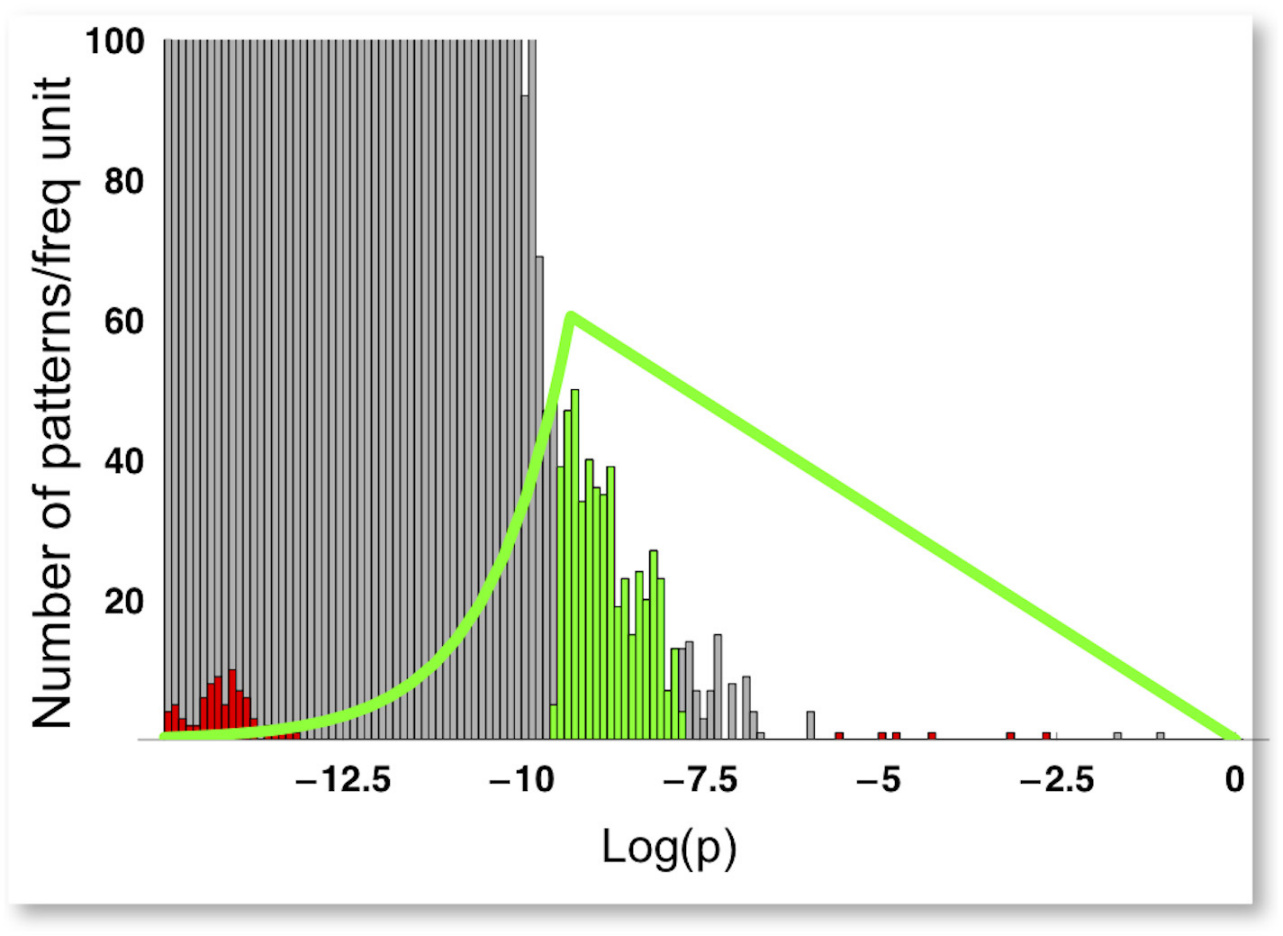
Patterns selection. The gray histogram represents the probability distribution, in natural log scale, of the 2^27^ possible 3x3 patterns, extracted from the dataset of RGB images used in this study [16]. The green curve is the model selection function with N = 500, W = 0.08. The green histogram is the probability distribution of corresponding selected patterns. The red histogram is the probability distribution of equiluminant patterns.

Our model explains the greater benefit of fine luminance information over color in rapid vision on general grounds, as a consequence of the statistics of natural scenes coupled with the limited resources of the visual system. Amongst other things, this implies that the specific chromatic coordinates used to encode the image should not affect the contribution of color to rapid vision. To test this, the model was rerun using a raw, CRT-based RGB color representation, instead of the MacLeod-Boynton representation. This avoids any possible influence from an *a priori* separation of chromatic and achromatic information. The results of our computations showed that none of the 39,366 possible equiluminant patterns, in the RGB representation, are recognized by our model as relevant features. The distribution of these patterns is indeed quite separated from the probability range predicted by our model for relevant patterns (Fig 6), and only a system with much larger computational resources would be expected to include them.

All of the features selected by our model contain both chromatic and luminance information, and it turns out that patterns with the same luminance structure are replicated in all possible colors. This further confirms that achromatic information is the only relevant and distinctive information used by the early visual system for feature detection and classification.

While these predictions are insensitive to the representation of the input image, the assumed representation of the output of the pattern selection inside the visual system does matter. In fact, the quantitative evaluation of the entropy is dependent on the specific assumption that the output of the compression stage takes the form of a pattern map (local features)–not just on the amount of assumed compression. Therefore, the observed concordance between the model and the psychophysical results presented here provides further support to a model of fast vision based on a lossy compression based on a pattern-filtering algorithm [9]. In sum, this study suggests that the computational limitations of the visual system have led to a system that at the finest spatial scales relies mostly on luminance, rather than color, for fast visual discrimination.

## Materials and Methods

### Ethic statement

The subjects of this study were aware of the purposes of the study and written consent forms were completed in accordance with the policy of the University of Chicago’s and University of Florence institutional review boards. The aforementioned review boards specifically approved this study. All data were analyzed anonymously.

### Image preparation

The study used a set of 560 calibrated natural images from a public online database [20], including animals, landscapes, foliage, fruits and flowers. Each pixel of these photographic RGB color images (768x576 pixel) was linearized [16], transformed into a LMS cone-based coordinate system using the Smith & Pokorny fundamentals [30], and then converted into MacLeod-Boynton color-space coordinates: *l* = L/L+M, *s* = S/L+M and *luminance* = L+M. After the coordinate system transformation, the bit depth of each pixel was reduced from 24 bits (corresponding to 2^24^ = 16,777,216 total color levels) to fewer bits as follows:

1. 1 bit total, assigned to the *l* coordinate: 2 color levels;
2. 1 bit total, assigned to the *luminance* coordinate: 2 gray levels;
3. 2 bits total, 1bit (least significant) assigned to *l* and 1 bit assigned to *luminance*
4. 2 bits total, both assigned to *luminance* (4 gray levels).

We limited our analysis to the L-M cone system since in primate retina L and M photoreceptors are more abundant than the S cones, that constitute about only 5–10% of the total cone population in the entire retina and are virtually non existent in the central region of the fovea [31]. Thresholds for digitization were chosen to preserve the maximum possible amount of information in the images, that is, they were chosen to maximize the average entropy flow from each image pixel. When only one coordinate is involved (either *l* or *luminance*), applying this criterion is equivalent to dividing the distribution at the median, (case 1, 2), or according to quartiles, (case 4). When two coordinates are involved (case 3), this criterion corresponded to finding the thresholds values T_x_ and T_y_ that satisfy the equation $\frac{\sum_{i}^{N}-p_{i}Log_{2}\left(p_{i}\right)}{\sum_{i}^{Q}-p_{i}Log_{2}\left(p_{i}\right)}$, where $p00=\int_{\begin{array}{l} x<Tx\\ y<Ty \end{array}}p\left(x,y\right)dx\;dy$, $p01=\int_{\begin{array}{l} x<Tx\\ y>Ty \end{array}}p\left(x,y\right)dx\;dy$, $p10=\int_{\begin{array}{l} x>Tx\\ y<Ty \end{array}}p\left(x,y\right)dx\;dy$ and $p11=\int_{\begin{array}{l} x>Tx\\ y>Ty \end{array}}p\left(x,y\right)dx\;dy$, and *p*_ij_ is the probability for each pixel to assume the values i, j. The solution to the above equations was found by numerical maximization.

For the last computational experiment, RGB original images were digitized according to $\underset{Tx,Ty,Tz}{Max}\left(\sum_{i,j,k\in \left\{0,1\right\}}^{}-p_{i,j,k}Log_{2}\left(p_{i,j,k}\right)\right)$, where $p000=\underset{\begin{array}{l} x<Tx\\ y<Ty\\ z<Tz \end{array}}{\int }p\left(x,y,z\right)dx\;dy\;dz,...p111=\underset{\begin{array}{l} x>Tx\\ y>Ty\\ z>Tz \end{array}}{\int }p\left(x,y,z\right)dx\;dy\;dz$.

### Extraction of visual filters and preparation of sketches

The procedure to extract entropy-optimal filters is similar to the one described in Del Viva et al. [9]. For each set of digitized images, the probability distribution of all possible 3x3 pixel patches was computed by considering all patches, centered on every pixel of the image (including overlaps). The probability distribution was evaluated by cumulating over all images of the set (Fig 6). In each condition tested, the optimal set of filters was selected as follows. Given the probability *p*_*i*_ of occurrence of each filter *i*, we assume that the system can select only a certain number N of filters with a maximum allowed total rate of acceptance *W*, such that Ʃ*p*_*i*_ *< W*. Under these constraints, the optimal performance of the filtering system is attained by using the selection function $f(p)\;=\;\frac{-\;p\text{ log}(p)}{\text{max}(1/N,\;p/W)}$, where–*p log(p)* is the entropy, and choosing the set of filters such that *f(p*_*i*_*)>c*, where the constant *c* is determined by the computational boundaries: $\int_{f(p)>c}^{}\delta (p)dp$ *<N* and $\frac{1}{N_{tot}}\int_{f(p)>c}^{}p\delta (p)dp$ *<W*, and δ*(p)* is the density of filters having probability of occurrence *p*, normalized to the total number *N*_*tot*_ of possible different filters. The optimal set of patterns will be then concentrated in a limited range of values of *p* around the maximum of *f(p)*, which occurs at *p = W/N*, and the range will therefore depend on both available storage size and bandwidth. The quantity $\frac{1}{N_{tot}}\int_{f(p)>c}^{}p\delta (p)dp$ is the average fraction of image elements that match successfully, and get preserved in the output—its inverse is the *compression factor* achieved by the filtering algorithm. Neglecting correlations, the total entropy of the set of N filters is $\sum_{i}^{\text{N}}-p_{i}\text{log(}p_{\text{i}}\text{)}$.

Fig 6 depicts the probability distribution of all possible patterns, the selection function and the distribution of selected patterns for the case of 3 bit RGB digitization reported below.

We set W = 0.05 as a constraint (a factor 20 compression), and we picked N = 50, as in previous 1-bit experiments [9].

For the RGB color space simulation, 3 bits were used, one for each coordinate. To allow for the expanded coordinate space, the model was set for a larger number of features (N = 500 out of 2^27^ = 134,217,728 possible patterns) and greater bandwidth (W = 0.08). *Luminance*, defined as R+G+B, could take only 4 possible discrete values (0,1,2,3), which could be encoded with 2 bits. “Equiluminant patterns” were defined as all patterns where all pixels shared the same luminance value; this included as a special case the class of uniform patterns, where all pixels had the same RGB value. The distribution of these equiluminant patterns is shown in red in Fig 6.

Sketches were derived from the same natural images of the database used in determining the filters sets and were obtained by keeping in the thresholded image only those 3x3 image patches corresponding to the chosen filter set. All possible 3x3 pixels patches, centered on every pixel of the image were considered (including overlaps). Some examples are shown in Fig 1d, 1e, 1h and 1i. The average information of sketches obtained with a certain set of patterns N is $\sum_{i}^{\text{N}}-p_{i}\text{log(}p_{\text{i}}\text{)}$. To compare the information of different sets we normalize this quantity by the average information carried by all possible patterns $\sum_{i}^{\text{Ntot}}-p_{i}\text{log(}p_{\text{i}}\text{)}$.

All simulations and computations were implemented with Mathematica software (Wolfram research) on MacBook Pro computers.

### Discrimination task

Sketches (23°x18° wide) were shown to the subjects in the center of the visual field, using a Sony Trinitron monitor (GDM-F520, refresh rate = 75Hz non-interlaced, resolution of 1360×1024 pixels, pixel size = 2 arcmin), controlled by a Macintosh G4 computer. In order to probe early stages of visual analysis, the presentation duration was 20 msec. [23].

The outputs from the R, G, and B guns of the CRT were linearized using a 10-bit lookup table. The spectral distribution of each of the three phosphors was measured at its maximal output using a PhotoResearch PR 650 spectroradiometer.

Sketches were followed by a mask of randomly colored pixels (duration 750 msec., 23°X18° wide), with the same luminances and color values as the sketch. Subsequently, two additional stimuli (19°x18° each) were presented side-by-side for 700 msec., one of them being the original (unfiltered) image corresponding to the sketch presented, and the other a distractor image, randomly selected from the same dataset. In each experimental condition, target, distractor, sketch and mask were digitized in the same way. The subject was asked to identify the image matching the sketch by pressing a computer key, according to a 2AFC procedure. In the control experiment, two equiluminant uniformly colored squares (18°x18° wide) were used, at the same chromaticities and luminances used in the first experiment. One of them was presented for 20 ms, followed by an equiluminant random-pixel mask (18°x18° wide, with same colors as the uniform patches) presented for 750 msec. Subsequently, the two equiluminant squares were presented side-by-side for 700 ms, and the subject asked to report which one was presented before. This was repeated after randomizing the chromaticity of the square in the first presentation.

Experiments 1, and 2 were performed by 3 naïve observers, interleaving different conditions; 5 naïve observers took part to experiment 3. All observers had normal color vision as assessed by Rayleigh matching using a Neitz anomaloscope.

Equiluminant sketches and digitized stimuli were prepared for each subject. Equiluminance was determined separately for each observer using heterochromatic flicker photometry (HFP) [32]. The HFP field size was 1.6° alternating at 12.5 Hz.

For chromatic stimuli two luminance values (7.58 and 13.47 cd/m^2^) and two chromaticities were used: *l* = 0.63, which appeared green and *l* = 0.70, which appeared magenta. *s* was arbitrarily set to 1.0 for EES “white”. When 1-bit color stimuli were used luminance was set to 7.58 cd/m^2^.

For achromatic stimuli luminance values were 7.58 and 13.47 cd/m^2^ for the 1 bit condition and 3.8, 7.58, 13.47 and 16.85 cd/m^2^ for the 2 bit condition.

Luminance (Michelson) contrast was 30% and color contrast was 5%. Viewing distance for all experiments was 60 cm. For each subject, at least 300 trials were presented for each condition tested.
